# Precision Medicine for Adolescent and Young Adult (AYA) Oncology

**DOI:** 10.3390/jpm13101449

**Published:** 2023-09-29

**Authors:** Amirrtha Srikanthan

**Affiliations:** 1 Division of Medical Oncology, The Ottawa Hospital, 501 Smyth Road, Ottawa, ON K1H 8L6, Canada; asrikanthan@toh.ca; Tel.: +1-(613)-737-7700; 2 Department of Medicine, Faculty of Medicine, University of Ottawa, 451 Smyth Road, Ottawa, ON K1H 8M5, Canada

Precision medicine, also referred to as “personalized medicine” is an approach in customizing disease prevention and treatment by integrating the unique differences found in individuals, typically identified through molecular or genomic characterization. Although the definition and scope have evolved over time, the goal of precision medicine is to ensure the right treatments are given to the right patients at the right time [1]. Most medical treatments are designed for the “average patient” using a one-size-fits-all approach, such as the use of nonspecific cytotoxic therapy against histology-specific tumors. This approach can be successful for some individuals but not for all. The approach of precision medicine is to tailor treatments in the hopes of increasing efficacy and reducing morbidity. The potential benefits of precision medicine are now clear, including improved diagnosis and monitoring, better identification of therapies that target a patient’s individual genomic profile or that of a target disease, reduced patient exposure to ineffective treatments, and lower cost from better targeting expensive therapies to those individuals most likely to benefit [1,2].

In the oncology setting, advances in precision medicine have already led to powerful new discoveries and approved treatments that are tailored to specific characteristics of individuals, such as a person’s genetic makeup, or the genetic profile of an individual’s tumor. Patients with a variety of cancers routinely undergo molecular testing as part of patient care, enabling physicians to select treatments that improve the chances of survival and reduce exposure to adverse effects [1]. Next-generation sequencing (NGS) technologies have evolved such that profiling of the mutational landscape of tumors can occur within relatively reasonable time frames and costs. This enables the integration of NGS into clinical care delivery [2]. In breast cancer, for example, the ability to distinguish actionable genomic alterations can lead to the tailored use of effective new therapies in the metastatic setting in a rationale and sequential manner, thereby prolonging survival and delaying the requirement for more toxic chemotherapy [3].

Adolescents and young adults (AYAs, aged 15–39 years) affected by cancer have unique treatment, survivorship, and palliation concerns compared to pediatric populations and older adults [4]. This demographic has historically suffered from poor survival gains and less access to supportive care and survivorship care [5]. In addition, given their age, they often face challenges that pediatric and older adult patients do not. AYAs with cancer tend to have better general health than older cancer patients but have a much longer life expectancy and face different obstacles than older patients during treatment and survivorship. AYAs with cancer face disruptions to their education and training, reintegration into school and vocational opportunities, disruption to key relationships (romantic and professional), increased financial burden, increased burden with disclosure around diagnosis, and increased risk of late treatment toxicities and secondary cancers. The late sequelae of anti-cancer therapy, and potential long-term side effects and recurrence risk, make survivorship care critical and more challenging for this demographic. Determining how to factor in these complex issues while protecting and maintaining one’s health is of great importance to AYAs with cancer and should be considered throughout AYA oncology clinical care.

Precision medicine for AYAs with cancer is a growing field that has the potential to have a positive impact on all facets of AYA oncology care from diagnosis to treatment and into survivorship. By focusing on the unique challenges that this age group faces, and tailoring treatments and supportive care to their unique needs, precision medicine can improve outcomes and reduce side effects, thereby impacting survival and quality of life. 

For example, AYAs with cancer have historically faced lower survival gains compared to pediatric or older adults with the same diagnosis [5]. Recognition of this survival gap has led to increased awareness and interest in understanding the biology of AYA cancer, from a tumor perspective and a host perspective. Using acute lymphoblastic leukemia (ALL) as an example, there is robust data demonstrating different outcomes in AYAs compared to children with ALL, and associations with basic biological features for why this may be the case [6]. In ALL, favorable genomic rearrangements (*ETV6-RUNX1*, *hyperdiploidy*) are more common in younger children than in AYAs, whereas unfavorable genetic abnormalities (*BCRABL*, *KMT2A *(*MLL*)**, *Ph-like*) increase among AYAs [5,6,7].

In addition to the underlying genomic changes of the cancer, AYAs themselves are more likely to have underlying genetic conditions that can contribute to higher chances of developing malignancies. Cancer predisposition syndromes such as Li-Fraumeni, hereditary nonpolyposis colorectal cancer, and neurofibromatosis 1 can all lead to the development of cancer when one is in the AYA age range [8]. Additionally, roughly half of AYAs with breast cancer aged less than 30 years have germline BRCA1, BRCA2, or TP53 mutations, which are associated with up to a 70% chance of developing breast cancer [9]. Precision medicine enables the recognition of these genetic variations to tailor screening and testing for these AYAs, in addition to offering medical interventions to prevent or treat cancer.

Precision medicine also allows for better understanding of how toxicity profiles differ in AYAs treated for cancer. Examples of toxicities more prevalent among AYAs include avascular necrosis (from prolonged steroid exposure), peripheral neuropathy (from vincristine), and hepatotoxicity (from asparaginase) [5]. These toxicities may be related to many factors, including variations in the metabolism of certain agents, the developmental stage of the individual receiving treatment, or the associated hormonal differences impacting metabolism. There are also examples of reduced toxicity, such as the better hematologic profiles seen in AYAs receiving alkylators compared with younger patients, which could represent a faster metabolism leading to potentially reduced exposure to these chemotherapy agents [5]. Understanding the mechanisms that influence these variations can lead to personalized treatment intensification or de-escalation to minimize toxicities while maintaining survival outcomes.

Tailored, patient-centered care for AYAs with cancer also applies to developing risk-based survivorship care [4]. Although robust data and survivorship care exists for childhood cancer survivors and certain older patients (such as with colorectal cancer and breast cancer), there are less data for patients diagnosed as AYAs. Additionally, models of care differ for AYAs versus pediatric populations and older adults, and programs that integrate survivorship care for AYAs are rare. There are emerging data demonstrating the negative late effects of cancer therapy on those diagnosed as AYAs, including second malignancies and cardiac toxicities, which result in increased mortality and morbidity [10]. Using precision medicine or personalized medicine to tailor follow-up recommendations to individual patients can serve to greatly enhance AYA care. Identifying biomarkers or pharmacogenomic changes in AYA cancer survivors that predict future morbidity or response to preventative treatments can help develop personalized survivorship care plans that improve quality of life and enable AYAs to continue attaining personal milestones.

Within all facets of AYA oncology care, precision medicine allows for clinical advances, whether by evaluating biomarkers at a genomic level or integrating novel management strategies that address unique AYA clinical presentations. Beyond precision medicine, AYA cancer care can be greatly improved by integrating various parameters, from clinical risk factors to sociodemographic factors, to holistically address a patient’s overall wellbeing. The advances in precision medicine fall under the paradigm of personalized, patient-centered care, and cannot be instituted in isolation. Concurrent with system-level changes to enhance AYA clinical care delivery and recognition of patient-level factors that influence health (such as socioeconomic status), precision medicine can improve AYA oncology care.
